# The who, when, and why of pacifier use

**DOI:** 10.1038/s41390-024-03540-6

**Published:** 2024-10-15

**Authors:** Kaloyan Mitev, Kelsey L. Frewin, Maria Augustinova, Paula M. Niedenthal, Magdalena Rychlowska, Ross E. Vanderwert

**Affiliations:** 1 Department of Psychology, University of Bath, Bath, England United Kingdom; 2 School of Psychology, University of East Anglia, Norwich, England United Kingdom; 3 Centre de Recherche sur les Fonctionnements et les Dysfonctionnements Psychologiques (CRFDP, UR 7475), Université de Rouen Normandie, Rouen, France; 4 Department of Psychology, University of Wisconsin-Madison, Madison, WI USA; 5 School of Psychology, Queen’s University Belfast, Belfast, Northern Ireland United Kingdom; 6 Cardiff University Centre for Human Developmental Science (CUCHDS), Cardiff University, Cardiff, Wales United Kingdom; 7 School of Psychology, Cardiff University, Cardiff, Wales United Kingdom

## Abstract

**Background:**

Social and familial consequences of pacifier use remain poorly understood. The present study attempts to shed more light on the characteristics of parents using pacifiers with their infants and to explore how pacifier use affects perceptions of infant emotionality, maternal stress, and parental efficacy.

**Methods:**

The study sample consisted of 428 mothers (range: 17–49 years) of infants (0–36 months) who completed a comprehensive questionnaire assessing infant and parent characteristics as well as parenting practices and pacifier use. We compared attitudes toward pacifiers, parenting stress, children’s levels of reactivity and self-regulation, and maternal efficacy among Pacifier Users, Never-Users, and families that Tried-Pacifiers.

**Results:**

The reported results reveal benefits of pacifier use for the family relationships, namely reduced parenting stress (*p* = 0.018), better parent-child dynamics (*p* < 0.001), and more positive perceptions of child’s affectivity (*p* = 0.006), which are all important aspects of infant development.

**Conclusion:**

Our findings highlight developmental benefits of pacifier use, a practice that is known to have both positive and negative long-term consequences for healthy child development. It is, therefore, important for health professionals to have discussions about the pros and cons of pacifier use with parents.

**Impact:**

- The study provides novel insights into how and why mothers use pacifiers and into the psychological consequences of this practice.
- We found pacifier use is associated with less maternal stress, better mother-child dynamics, and more positive perceptions of child’s affectivity.
- Our findings document developmental benefits of pacifier use adding a new perspective to the debate on pacifiers.
- Doctors, health visitors, pediatric nurses, and midwives may consider this evidence when advising parents on pacifier use.

## Introduction

Pacifiers have accompanied humanity for more than 3000 years, as evidenced by excavations in Italy, Cyprus, and Greece.^1^ Pacifiers are now used across the world to soothe fussy or colicky babies, reduce the pain of teething, and promote restful sleep.^2^ Widespread popularity of pacifiers is reflected in empirical evidence documenting the benefits of non-nutritive sucking^3,4^ including pain management in neonates,^5,6^ shorter duration of hospital stays,^7,8^ and greater readiness for bottle feeding in preterm infants.^8^ Despite these benefits, pacifier use remains a controversial parental practice. While pacifier use is recommended for preventing sudden infant death syndrome (SIDS),^9,10^ it has also been linked with acute otitis media and other infections,^11–14^ dental malocclusion,^15^ and excessive weight gain.^16^

In addition to the mixed health consequences, the psychological consequences of pacifier use remain poorly understood.^17–20^ Niedenthal and colleagues^21^ suggest that the diurnal use of pacifiers in infancy decreases infants’ spontaneous imitation of others’ facial expressions (i.e., facial mimicry) as well as their empathy and emotional intelligence at later developmental stages. However, the correlational nature of this research makes it challenging to tease apart the causes and consequences of this parental practice. For example, pacifiers could be used by caregivers with children considered more difficult (i.e., children whose negative emotions are challenging to manage).^22^ Alternatively, seeing the pacifier in an infant’s mouth could reduce the extent to which adults imitate the baby’s facial expressions.^23^ This behavior is involved in the development of babies’ own facial mimicry and emotional competence.^20,21^

In addition to infant temperament, the characteristics of parents may also play a role in pacifier adoption. For example, stressed caregivers might be more inclined to introduce pacifiers. Moreover, existing research shows associations between pacifier use and maternal anxiety and rigid breastfeeding styles.^22^ Beyond these traits, little is known about the characteristics of parents giving pacifiers to their infants. Here we recruited a large sample of British parents to examine demographic profiles of pacifier users versus non-users. Among these groups, we compared the frequency and circumstances of pacifier use and relations between the use of pacifiers and parenting stress as well as infant temperament. We also examined beliefs about pacifiers among users and non-users.

## Method

### Participants & procedure

The study had a cross-sectional questionnaire design. Participants were 428 mothers (*M* = 33.27 years, *SD* = 5.63, range: 17–49 years) of infants between 0 and 36 months of age (*M* = 18.33 months, *SD* = 9.37, 205 male, 221 female, & 2 non-responses). Respondents were recruited via Pureprofile, an online market research company that pays subscribers to complete questionnaires. Participants underwent identity validation, IP checks, and provided demographic information including parenting status as part of registering their Pureprofile accounts. A total of 1740 residents of the United Kingdom received a daily email from Pureprofile and started the questionnaire. However, 1090 failed to complete the survey or did not correctly answer three attention screening questions (e.g., *If you live in the United Kingdom please select Strongly Disagree*), 13 were over the age of 50 years, and 209 were fathers. About half the sample reported using a pacifier now or in the past (*n* = 243; 56.8%).

After clicking the link, participants provided consent and started the survey implemented in Qualtrics (Provo, UT). Upon completion of the questionnaire, which took approximately 25 min, participants were thanked, debriefed, and paid. The project received approval from the School of Psychology Ethics Committee at Cardiff University.

### Materials

The study questionnaire assessed infant and parent characteristics as well as different aspects of pacifier use and parenting practices. We describe parts of the survey relevant to the present research below.

At the beginning of the questionnaire, the parents provided demographic information about their baby (sex, age, siblings) and themselves (role: mother/father, age, education, primary language). To report their highest educational degree, participants selected the appropriate answer among 6 options (*primary*, *GCSE/O Levels*, *A Levels/BTEC*, *Bachelor’s Degree*, *Master’s Degree*, *Doctorate*). They were then asked if their baby used a pacifier (commonly referred to as a *dummy* in the UK) currently or in the past (*yes/no* response options). Participants who reported not using a pacifier were asked if they introduced it, but their baby refused to use it. Participants who reported using a pacifier were presented with additional questions. One of them examined the frequency of pacifier use and had 6 response options: *more than 3 times a day, 2-3 times a day, daily, 2-3 times a week, about once a week, occasionally*. Participants also answered five questions about the person who influenced their decision to use the pacifier. Specifically, they were asked if a member of their family suggested that the infant should use a pacifier (3 response options: *yes/no/not applicable*), who this person was, or whether a friend or a physician made the recommendation (*yes/no*). Finally, participants rated the extent to which the person influenced their decision to use a pacifier and answered on a scale ranging from 1 (*not at all*) to 5 (*very much*).

Anxiety and self-efficacy of the mother were also assessed. Anxiety traits were measured using the Spielberger Trait Anxiety Inventory^24^ (e.g. *I feel pleasant; I lack self-confidence*). Scores range from 20 to 80. We used the Maternal Self-Efficacy Scale^25^ to assess how good a mother feels in different situations with their baby (e.g., *How good are you at getting your baby to have fun with you?*) rated on a 1 (*No good at all*) to 4 (*Very good*). Scores range from 10 to 40.

We measured parenting stress using the Parenting Stress Index – Short Form.^26^ This questionnaire is comprised of 36 items rated on a Likert-type scale ranging from 1 (*strongly disagree*) to 5 (*strongly agree*) and was used to evaluate and identify problems in the child’s or parent’s behavior. It is divided into three subscales: Parental Distress, Parent-Child Dysfunctional Interaction, and Difficult Child (12 items each). Subscale scores range from 12–60 with higher values representing more stress.

Infant temperament, defined as a child’s reactivity and self-regulation shaped by heredity and experience, was measured by the Infant Behavior Questionnaire Revised - Very Short Form.^27^ The parent-report questionnaire is comprised of 37 items and participants indicated their responses using Likert scales ranging from 1 (*never*) to 7 (*always*) to report how often their baby displayed certain behaviors over the last week (e.g., *During a peekaboo game, how often did the baby laugh?*; *When tired, how often did your baby show distress?*). Items are grouped into 3 subscales: Positive Affectivity (13 questions), Negative Affectivity (12 questions) and Regulation Capacity (12 questions). Scores for each subscale are averaged and range from 1 to 7, with higher values representing higher levels of each dimension.

All participants completed a 6-item measure of their attitudes toward pacifiers. Three items concerned positive attitudes (e.g., *dummies reduce a baby’s distre*ss), and three concerned negative attitudes (e.g., *I have negative feelings toward the use of dummies*). Participants answered on scales ranging from 1 (*strongly disagree*) to 5 (*strongly agree*). This scale showed strong internal consistency (α = 0.73). Mothers also rated how frequently they would offer their babies a pacifier in ten social contexts (e.g., *when s/he is asleep or trying to fall asleep*; *when other kids are present*, see Table 2 for all items) on a scale ranging from 1 (*never*) to 5 (*very frequently*).

### Statistical analysis

We compared the characteristics of parents and infants who used pacifiers with those who did not use them. Mothers whose children used a pacifier at the time of the study or in the past were coded as Pacifier-Users and those who had never used a pacifier, as Non-Users. We aimed to characterize how pacifiers are used and examine qualitative differences between Users and Non-Users.

Although this binary categorization was used in most analyses, when examining attitudes towards pacifiers, we assessed three categories: Pacifier-Users, Never-Users, and families that Tried-Pacifiers. The categories were created based on the question *Did you introduce a dummy but your baby refused to use it?* Those who answered negatively were coded as Never-Users (*n* = 128), and those who answered positively as Tried-Pacifiers (*n* = 57). Analyses were conducted in SPSS v27 and we adjusted all post-hoc tests using the Bonferroni correction.

## Results

### Pacifier-users tend to be less educated than non-users

We examined whether child or maternal characteristics differed between Pacifier-Users and Non-Users (see Table 1). The groups did not differ on the sex or age of the child, maternal age, anxiety, or self-efficacy (all *p*s > 0.16). Mothers who had a high school diploma or lower educational degree were more likely to use a pacifier compared to mothers with a Bachelor’s degree or postgraduate training (χ^2^(1) = 18.88, *p* < 0.001).

**Table 1:** Demographic characteristics by pacifier use.

Demographics	Users	Non-users
Infant sex (*N* = 426)^a^		
Boys	27.9% (*n* = 119)	20.2% (*n* = 86)
Girls	28.9% (*n* = 123)	23.0% (*n* = 98)
Infant age (*N* = 428)^a^		
0–1 years	15.0% (*n* = 64)	9.8% (*n* = 42)
1–2 years	27.8% (*n* = 119)	20.1% (*n* = 86)
2–3 years	14.0% (*n* = 60)	13.3% (*n* = 57)
Maternal age (years) (*N* = 414)^b^	33.26 (*SD* = 5.89)	33.28 (*SD* = 5.29)
Maternal education (*N* = 428)^a^		
High school diploma or less	30.4% (*n* = 130)	14.0% (*n* = 60)
Bachelor’s degree or more	26.4% (*n* = 113)	29.2% (*n* = 125)
Maternal anxiety (*N* = 424)^b^	43.63 (*SD* = 11.10)	43.29 (*SD* = 11.03)
Maternal self-efficacy (*N* = 428)^b^	3.33 (*SD* = 0.37)	3.28 (*SD* = 0.35)

Due to missing data, the sample sizes that were included in the analyses are displayed next to each demographic characteristic.

^a^*χ*2-test was used to compare groups. Sample percentages and count are reported.

^b^t-test was used to compare groups. Sample means (standard deviations) are reported.

### Pacifier-users use them frequently

Within the Pacifier-Users group, 33.0% (*n* = 77) reported using the pacifier more than 3 times a day and 25.3% (*n* = 59) reported using a pacifier 2 to 3 times a day. Daily users accounted for 15.5% (*n* = 36) while 3.4% (*n* = 8) used it 1 to 3 times a week. Only 22.7% (*n* = 53) used it less than once a week (occasionally). When asked about the contexts in which they typically use a pacifier (see Table 2 for details), most mothers reported using a pacifier at bedtime or to comfort their crying baby. Pacifiers were used less frequently in social contexts involving friends, family, and other children.

**Table 2:** Frequency of pacifier use in ten contexts.

When s/he is asleep or trying to fall asleep.	4.21 (1.28)
When there is nothing else that would make my baby stop crying.	3.76 (1.37)
When I am travelling (e.g., in a car, on a bike) with him/her and there is no one else with me.	3.07 (1.50)
When I am in a public place (e.g., waiting in a line, grocery shopping) and my baby needs to be quiet.	2.85 (1.50)
When s/he is awake outside of the home with strangers around.	2.37 (1.36)
When I want to prevent my baby from sucking on his/her thumb.	2.34 (1.47)
When s/he is awake outside of the home with family/friends around.	2.32 (1.32)
When s/he is awake at home with me or other family members attending to him/her.	2.28 (1.27)
When I am alone with him or her and I don’t want to be disturbed.	2.26 (1.41)
When other kids are present (e.g., when she/he is at the park, in daycare).	2.02 (1.21)

Ratings were from 1 “Never” to 5 “Very Frequently. Numbers displayed are the mean ratings with standard deviations in parentheses.

### Pacifiers are rarely recommended by physicians

When the participants were asked whether anyone had suggested they give their baby a pacifier 28.1% (*n* = 64) said it was a family member, 16.2% (*n* = 37) indicated it was a friend, and only 10.1% (*n* = 23) received this advice from a physician. Recommendations did not strongly influence mothers’ decisions to use a pacifier (*M* = 2.56; *SD* = 1.16 out of 5). Most mothers (62.3%; *n* = 142) reported not receiving a recommendation.

### Pacifier-users report less parenting stress

**Fig. 1:**
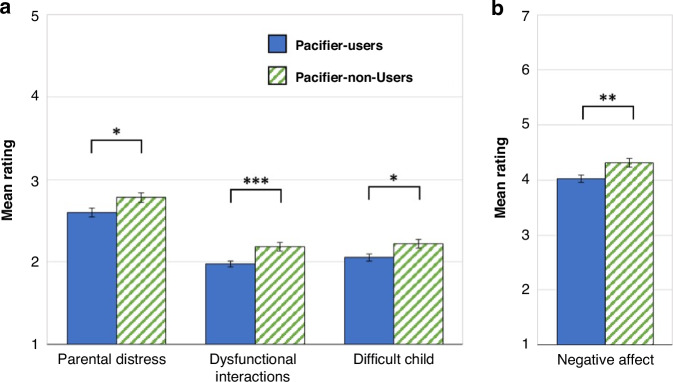
Differences between Pacifier-Users and Non-Users on their ratings of **a** parenting stress (Parenting Stress Index – Short Form) and **b** the negative emotionality of their child (Infant Behavior Questionnaire Revised - Very Short Form). ***p < 0.001, *p < 0.05.

We used *t*-tests to compare the Parenting Stress Index subscales of Parental Distress, Parent-Child Dysfunctional Interaction, and Difficult Child across pacifier groups. Pacifier-Users reported significantly lower Parental Distress (*M* = 2.60, *SD* = 0.78) than Non-Users (*M* = 2.78, *SD* = 0.81; *t*(426) = 2.37, *p* = 0.018, *d* = 0.232); significantly less Dysfunction in Interactions than Non-Users (Pacifier-Users: *M* = 1.98, *SD* = 0.57; Non-Users: *M* = 2.19, *SD* = 0.69; *t*(426) = 3.43, *p* < 0.001, *d* = 0.335); and perceived their child as less Difficult (Pacifier-Users: *M* = 2.06, *SD* = 0.69; Non-Users: *M* = 2.22, *SD* = 0.76; *t*(426) = 2.35, *p* = 0.019, *d* = 0.229; Fig. 1a). These differences remained significant (Dysfunction in Interactions, *F*(1424) = 6.45, *p* = 0.01, η2 = 0.015) and marginally significant (Parental Distress, Difficult Child, *F*(1424) = 3.69, *p* = 0.05, η2 = 0.01 and *F*(1424) = 3.20, *p* = 0.07, η2 = 0.007, respectively) after controlling for dichotomized parental education (high school diploma or lower vs. Bachelor’s degree or postgraduate training).

### Pacifier users report less negative affect in their child

The Infant Behavior Questionnaire Negative Affectivity scale measured, via maternal report, the infant’s propensity to experience negative emotions. Pacifier-Users reported less Negative Affect (*M* = 4.02, *SD* = 1.06) compared to Non-Users (*M* = 4.31, *SD* = 1.11; *t*(424) = 2.74, *p* = 0.006, *d* = 0.268; Fig. 1b). This difference remained significant after controlling for parental education, *F*(1422) = 4.39, *p* = 0.04, η2 = 0.01. There were no differences between Pacifier-Users and Non-Users on Positive Affectivity or Regulation Capacity.

### Experience with pacifiers shapes more positive attitudes towards pacifiers

**Fig. 2:**
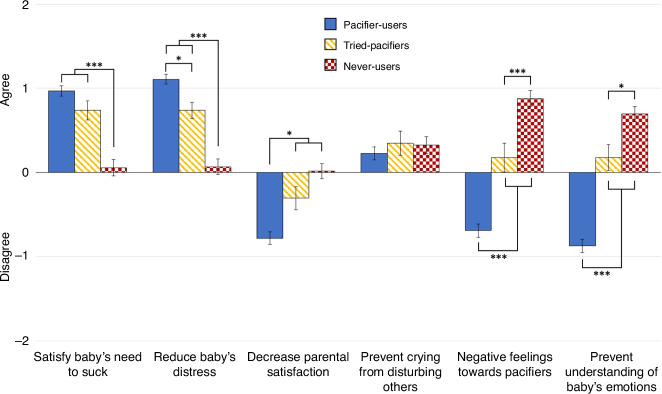
Beliefs about pacifiers. The figure displays ratings of agreement with statements about beliefs and purposes of pacifiers by mothers who are previous or current Pacifier-Users (solid blue), mothers who Tried-Pacifiers (striped yellow) but their baby rejected them, and mothers who are Never-Users (checkered red). For ease of interpretation, *neither agree nor disagree* responses were set to zero.

To better understand the mother’s attitudes towards pacifiers, we examined the perceptions and beliefs of Pacifier-Users, those that Tried-Pacifiers, and Never-Users. We used univariate ANOVAs with endorsement of each of the 6 beliefs as the dependent variable and group as the fixed factor. Figure 2 presents a summary of the findings for each belief.

Participants’ endorsement of the item: *Pacifiers satisfy a baby’s need to suck* significantly differed by group *F*(2427) = 35.88, *p* < 0.001; η^2^ = 0.144. Pacifier-Users and Tried-Pacifiers groups did not differ significantly (*t*(427) = 1.60, *p* = 0.333), but endorsed this item more compared to Never-Users (*t*(427) = 8.45, *p* < 0.001 and *t*(427) = 4.32, *p* < 0.001, respectively).

Endorsement of the item: *Pacifiers reduce a baby’s distress* significantly differed by group *F*(2427) = 45.06, *p* < 0.001; η^2^ = 0.207. Pacifier-Users endorsed this item more frequently than the Tried-Pacifiers group (*t*(427) = 2.78, *p* = 0.017). Both groups endorsed this item more than the Never-Users (*t*(427) = 10.58, *p* < 0.001 and *t*(427) = 4.66, *p* < 0.001, respectively).

Endorsement of the item: *Pacifiers decrease parental satisfaction with the time they spend with their baby* significantly differed by group *F*(2427) = 27.43, *p* < 0.001; η^2^ = 0.095 where Pacifier-Users disagreed more frequently than Tried-Pacifiers (*t*(427) = 2.94, *p* = 0.010) and Never-Users (*t*(427) = 6.55, *p* < 0.001) but Tried-Pacifiers and Never-Users did not differ in their level of disagreement (*t*(427) = 1.77, *p* = 0.230).

Endorsement of the item: *Pacifiers prevent a baby’s crying from disturbing other people* did not differ across groups *F*(2,427) = 0.409, *p* = 0.665; η^2^ = 0.002.

Endorsement of the item: *I have negative feelings towards pacifiers* significantly differed by group *F*(2427) = 73.32, *p* < 0.001; η^2^ = 0.257 where Pacifier-Users disagreed more than the Tried-Pacifiers group (*t*(427) = 4.88, *p* < 0.001) and Never-Users (*t*(427) = 11.98, *p* < 0.001). And Never-Users endorsed this item more than Tried-Pacifiers group (*t*(427) = 3.70, *p* < 0.001).

Endorsement of the item: *Pacifiers prevent me from understanding my baby’s emotions* significantly differed by group *F*(2427) = 86.84, *p* < 0.001; η^2^ = 0.290 where Pacifier-Users disagreed more than the Tried-Pacifiers group (*t*(427) = 6.31, *p* < 0.001) and Never-Users (*t*(427) = 12.81, *p* < 0.001). Further, Never-Users endorsed this item more than Tried-Pacifiers group (*t*(427) = 2.94, *p* = 0.010).

Overall, these analyses show that mothers who used, or tried using, a pacifier with their baby, had more positive beliefs and attitudes towards pacifiers. In contrast, mothers who never used a pacifier endorsed more negative beliefs about pacifier use.

## Discussion

This is the first comprehensive study examining the practice, perceptions, and psychological correlates of pacifier use by mothers. In the current study, more than half of the mothers gave a pacifier to their baby, a consistent trend reported for the Western part of the world.^28–30^ There were no differences between age of mother or age or sex of the infant when choosing to use a pacifier. Additionally, pacifier use did not differ based on maternal anxiety or parenting self-efficacy. Despite this general similarity in psychological characteristics, pacifier users tend to be less educated than non-users – suggesting a possible role of socio-economic status in decision-making around pacifier usage.^30^ We found that when pacifiers are used, mothers use them frequently throughout the day to help calm their baby, help their baby to sleep, or when their concentration is away from their baby (e.g., when driving or shopping). Mothers see pacifiers as useful when babies are asleep or trying to sleep, when there is nothing else to help their baby stop crying, and during moments of divided attention when caring for their infant alone, rather than using them indiscriminately. Indeed, these behaviors are consistent with the baby-friendly hospital recommendations^31^ and research highlighting the benefits of non-nutritive sucking for the prevention of SIDS, calming, and aiding sleep.^3,4,9,10^ In contrast, pacifiers were least frequently used when the baby was outside of the home with family or friends, to prevent the baby sucking its thumb, or when the baby was awake and outside the home with strangers around.

Pacifier use was related to lower levels of parenting stress. Mothers who use pacifiers report less stress and more positive interactions with their child relative to pacifier non-users. Not surprisingly, mothers who use pacifiers also perceive their child as less difficult and displaying less negative affect compared to pacifier non-users. Reducing parenting stress and optimizing the parent-infant dynamic can have significant benefits to child outcomes. Research has shown that decreased parental stress during infancy is associated with improved child emotion regulation, social competence, and achievement at 4 years^32,33^ and decreased disruptive behaviors at 5 years.^34,35^ More positive parent-infant interactions were also linked with better language production at 4 years^36^ and better academic achievement in middle school.^37^ Therefore, pacifiers may help support positive parenting practices that are known to have long-term benefits to the child across developmental domains.

Compared to physicians, it was more common for family or friends to recommend pacifiers to mothers, consistent with evidence reported by Mauch and colleagues^28^. Given the health benefits and risks of pacifier use^9,11,16,22^ and the potential risk of misinformation from non-expert sources; doctors and other health professionals should consider talking with parents about pacifiers at early check-ups, particularly when parents express distress or difficulty dealing with their infant’s negative emotions. Such conversations should include advice for the cessation of pacifier use by 2 years.^11,17^

Attitudes surrounding pacifier use were consistently split between pacifier users and never-users. Pacifier users agreed with statements surrounding the utility of pacifiers in non-nutritive sucking and reducing negative emotions in their baby, whereas they disagreed that pacifiers intruded on their ability to parent. Conversely, never-users reported more negative feelings about pacifiers and felt pacifiers may prevent their understanding of their infants’ emotions. Mothers that had tried a pacifier shared agreement with pacifier users on the utility of pacifiers and remained more agnostic about other aspects of pacifiers. These findings suggest that mothers consider multiple factors in their decision on whether or not to use a pacifier. We did not probe mothers’ reasoning for their negative attitudes which may arise from socio-cultural, familial, or the prevalence of pseudo-evidence suggesting pacifiers can disrupt breastfeeding.^8,9^ Future research on mothers’ attitudes surrounding pacifier use should probe the specific negative attitudes held and the sources informing those attitudes.

Our study highlights potentially important benefits of pacifier use in early infancy, but those findings should be considered with a few caveats. Our sample included a large proportion of mothers who have or have not given their infant a pacifier. As a result, the psychological traits we assessed may reflect characteristics of mothers who chose to use pacifiers, rather than a direct outcome of using pacifiers. However, the broad disagreement with the statement ‘*pacifiers decrease parental satisfaction*’ increases confidence that pacifiers reduce stress and improve mother-infant interactions rather than the other way around. The same argument can be made for the negative emotionality of the infants because causality cannot be established without assessing that trait before the introduction of the pacifier. The study is an online questionnaire conducted with an external market research company. While Pureprofile aims to recruit representative samples, our participants needed to have access to a computer or a mobile device and time to complete the survey. Therefore, our findings should be replicated using different recruitment methods and with different participant groups, ideally accounting for other factors such as socioeconomic status, family dynamics, or cultural differences in pacifier use and beliefs about pacifiers. Finally, the present study relies on maternal report for all the measures of both maternal and infant characteristics. Our focus was on parents’ perceptions of pacifier use and so this limitation has less of an impact. However, behavioral assessments of mothers’ and infants’ emotional competence would provide more objective descriptions of these domains, shedding more light on the long-term consequences of pacifier use.

The debate surrounding whether or not to provide pacifiers to infants will likely continue. Importantly, research suggests a wide range of positive applications for pacifiers,^3,4,9,31^ but less is known about the impact of pacifiers on the long-term development of language, cognitive skills, emotion regulation, and parent-child relationships. Future research is needed to address these questions, highlighting the ages when pacifiers increase risk for developmental delays in these domains and providing better guidance for cessation of pacifier use.

## Conclusions

Previous research tended to explore controversial aspects of pacifier use with little acknowledgement of the potential benefits. Here, we show that the long history of pacifier use in humans might be related to immediate benefits to the mother and infant. We found that mothers who use pacifiers report less negative affect in their infant, less stress as a parent, and more positive interactions with their infant relative to mothers who do not use pacifiers. These important aspects to the mother-infant relationship have long-term consequences for the healthy development of the child. It is important that health professionals are aware of these potential benefits and consider them when discussing pacifier use with parents.

## Data Availability

All data presented in this manuscript are available upon request from the authors for research purposes. Requests may be sent to Dr. Magdalena Rychlowska (corresponding author).
